# Ischemia Time Impacts on Respiratory Chain Functions and Ca^2+^-Handling of Cardiac Subsarcolemmal Mitochondria Subjected to Ischemia Reperfusion Injury

**DOI:** 10.1186/1749-8090-10-S1-A126

**Published:** 2015-12-16

**Authors:** Marcus Leistner, Stefanie Sommer, Peer Kanofski, Christian Moch, Christoph Schimmer, Ivan Aleksic, Rainer Leyh, Sebastian-Patrick Sommer

**Affiliations:** 1 Department of Cardiothoracic Surgery, Wuerzburg University Hospital, 97080 Wuerzburg, Germany

## Background/Introduction

Significant mitochondrial function impairment is known to result from cardiac ischemia reperfusion injury (IR) precipitated by cardiopulmonary bypass during heart surgery.

## Aims/Objectives

We sought to determine the effect of different ischemia time spans in cardiac IR on mitochondrial respiratory chain (RC) function, inner membrane polarization and Ca2+homeostasis.

## Method

Wistar rat hearts were harvested and divided into 4 groups of stop-flow induced warm global IR: 0, 15, 30 and 40 min of ischemia followed by 30 min of reperfusion, respectively. Myocardial contractility was determined from left ventricular pressure records (dP/dt, dPmax). Subsarcolemmal mitochondria (SSM) were isolated and analyzed regarding electron transport chain (ETC) coupling using a Clark-type electrode (polarography), membrane polarization (JC1 fluorescence) and Ca2+-handling in terms of Ca2+induced swelling and Ca2+-uptake and release (Ca2+-sensitive electrode).

## Results

IR in general depressed LV contractility irrespective of ischemia duration. In contrast, increasing length of ischemia time highly significantly promoted ETC uncoupling at complex I-V and II-IV in state 3 respiration, respectively. Membrane potential showed a distinct hyperpolarization in IR30/30 and IR40/30 compared to the other groups (p < 0.0001), continuously wearing off after CCCP-induced uncoupling. Regarding Ca2+-induced swelling, light transmission of IR40/30 SSMs started to differ significantly (p < 0.04) from IR0/30 after 6.5 min of Ca2+-addition, swiftly followed by IR15/30 (8.5 min) and 30/30 (16.5 min). All effects were delayed by app. 3.6 min by pyruvate addition in parallel assays also halving recorded swellings. Ca2+-uptake revealed slower rates and greater spans in IR15/30 and IR30/30 (p < 0.005) whereas Ca2+-release was delayed for ischemia an duration ≤30 min (p < 0.0001).

## Discussion/Conclusion

Longer ischemia duration in IR injury gradually impairs SSMs in terms of respiratory chain function and Ca2+-homeostasis. Membrane hyperpolarization appears to be responsible for impaired Ca2+-cycling and ETC function. Therefore, ischemia time should be considered an important factor influencing IR experiment-derived conclusions.

